# Human Pathogenic Bacteria Within the Nasal and Rectal Microbiome of *Macropus giganteus*

**DOI:** 10.3390/tropicalmed10110322

**Published:** 2025-11-17

**Authors:** David Arroyo, Amy Peart, Brian Vesely, Andrew Trudgian, Jessica Chellappah

**Affiliations:** 1 Australian Defence Force Malaria and Infectious Disease Institute, Enoggera, QLD 4051, Australia; 2 Microbiology Department, Melbourne Pathology, Collingwood, VIC 3066, Australia; 3 Walter Reed Army Institute of Medical Science-Armed Forces Research Institute of Medical Sciences (WRAIR-AFRIMS), Bangkok 10400, Thailand; 4 School of Public Health, University of Queensland, Herston, QLD 4006, Australia

**Keywords:** *Staphylococcus*, skin and soft tissue infections, zoonosis, eastern grey kangaroos, one health

## Abstract

This study represents the first investigation into the prevalence of pathogenic bacteria in isolated, free-ranging Eastern Grey Kangaroos (*Macropus giganteus*) inhabiting a human-shared environment. Samples were collected from the nasal and rectal passages of state-authorised culls of *M. giganteus* within a military training area, where recruits had reported recurrent cases of skin and soft tissue infections. The objective was to identify clinically relevant pathogenic microorganisms present in the nasal and rectal flora of these kangaroos. Analysis revealed carriage rates of 11% for methicillin-sensitive *Staphylococcus aureus* (MSSA) and 2% for methicillin-resistant *S. aureus* (MRSA). Other potentially pathogenic bacteria isolated included *Pseudomonas* spp., *Streptococcus* (Groups B and D), *Acinetobacter* spp., and multiple coagulase-negative *Staphylococcus* (CoNS) species. Notably, CoNS species were present in 17% of nasal isolates, with *Mammaliicoccus sciuri* (formerly *Staphylococcus sciuri*) detected in 41% of these isolates, suggesting a potential reservoir for antibiotic resistance genes and virulence factors. These findings support a One Health perspective, highlighting the interconnectedness of pathogenic bacteria, *M. giganteus*, humans, and their shared environment.

## 1. Introduction

*Staphylococcal aureus* (*S. aureus*) is a Gram-positive, environmentally ubiquitous, facultative anaerobic bacterium commonly found as a commensal microorganism within the nasal passages, throat, mucous membranes, and skin of both humans [1] and animals [2]. Worldwide, *S. aureus* is responsible for over 1-million deaths per year [3], and remains the most common pathogen implicated in human skin and soft tissue infections (SSTIs) regardless of geographic region [4]. Resulting skin infections can range from simple, superficial infections to more serious skin infections such as cellulitis or necrotising fasciitis. Additionally, *S. aureus* has the potential to become invasive, resulting in severe and life-threatening infections such as infective endocarditis, necrotising pneumonia, and meningitis, which are also potential sequelae of *S. aureus* infection. *S. aureus* can be transmitted through fomites or direct contact, spreading between the environment, animals, and humans [1,5], and can survive on surfaces for months [6]. Antibiotic-resistant *S. aureus* has become a significant public health threat, as evidenced by the emergence of methicillin-resistant *S. aureus* (MRSA), which has increased in prevalence over time and has high morbidity and mortality rates [7,8,9,10,11]. Populations living in proximity, such military personnel, are at a high risk of staphylococcal SSTIs [10,12]. From 2008 to 2015, SSTIs represented 10% of all medical consultations for American soldiers, with over 429,000 diagnoses recorded [12]. Basic training remains a high-risk period for the development of SSTIs, with *S. aureus* identified as the predominant causative agent [12]. This is a result of their unique living and training environment, which involves residing in close living quarters, temperature extremes, limited time for personal hygiene, close interaction with each other and the environment, and high rate of skin trauma due to vigorous training. Incidence and outbreaks of SSTI caused by MSSA and MRSA among Australian Army Training Recruits have been investigated [13,14].

The family *Macropodidae*, which includes approximately 67 species, including kangaroos, wallabies, and quokkas, are native to Australia and Papua New Guinea. Although literature is limited, *S. aureus* has been identified as a pathogen in Eastern Grey Kangaroos [15]. The potential zoonotic transmission of *S. aureus* from these animals to humans, particularly in regions where human and kangaroo populations frequently intersect, is concerning. Macropods frequently inhabit regions used by the Australian Defence Force for training, which typically involves close contact with the shared environment raising questions surrounding the role that these animals play in *S. aureus* transmission. The prevalence of *S. aureus* strains colonising macropods carrying genes of clinical significance to humans such as those that code for enterotoxins, exfoliative toxins, epidermal cell differentiation inhibitors, toxic shock syndrome toxin, and the Panton–Valentine leucocidin toxin remains unknown.

Research by Chen et al. on three species of free-ranging wallabies (*Petrogale xanthopus*, *Petrogale lateralis*, and *Macropus eugenii*) in South Australia detected nasal colonisation with antibiotic-resistant coagulase negative staphylococci, non-antibiotic-resistant *S. aureus*, and multidrug-resistant Staphylococcal species, including *S. aureus* [16]. This indicates that these bacteria are commensal microorganisms within the wallaby biome and suggests that non-pathogenic staphylococcal species may be susceptible to acquiring antibiotic resistance genes over time. A follow-up study of the same staphylococcal isolates discovered that there was transmission of MSSA between humans and wallabies [17]. The aim of the current study was to isolate bacteria with human pathogenic potential from the nasal and rectal microbiomes from recently culled Eastern Grey Kangaroos (*Macropus giganteus*) frequenting the military area in which ongoing incidence of SSTI in military recruits are being reported and investigated [14].

## 2. Methodology

### 2.1. Investigated Area

In April 2024, a scheduled state-licenced kangaroo cull was conducted at a military area used for training by The Australian Army School of Infantry. Our team was permitted access within 4 h post-cull by state-authorised contractors.

### 2.2. Population Characteristics

The Eastern Grey Kangaroos inhabiting this region have never been domesticated, are not human-fed, and roam the base freely without any direct human contact. The area is secured, and land animal movements are closely monitored by Range Control Officers. Kangaroos are not known to migrate in and out of the military area and are likely representative of the population that was present during the incidence of SSTI among training recruits in 2022 [14].

### 2.3. Macropus Giganteus Baseline Health Assessment

Animals were visually assessed by a trained zoologist for signs of acute or chronic illness although this was not a compressive assessment. There were no disease outbreaks among the kangaroos on base.

### 2.4. Collection of Samples

Both nasal and rectal/peri-anal swabs were collected from 100 culled Eastern Grey kangaroos within 4 h of culling and all swabs [Amies Agar Gel Transport Swabs] were processed within 48 h of collection. In consultation with three zoologists, carcasses were relocated less than 500 m from the culling site. Expert opinion indicated that the process used for this study would not alter the post-mortem microbiota. All carcasses were handled and transported with care to prevent contamination from environmental flora.

### 2.5. Sample Analysis

Selective media were implemented to isolate clinically relevant bacteria using MSSA/MRSA plates, extended-spectrum beta-lactamase (ESBL) plates, Columbia agar with colistin and nalidixic acid (CNA), and MacConkey agar plates. Subsequent bacterial identification and characterisation was performed by Melbourne Pathology using matrix assisted laser desorption ionisation-time of flight (MALDI-TOF) mass spectrometry.

### 2.6. Ethics

Ethics approval was obtained from the Department of Defence and Veteran’s Affairs Human Research Ethics Committee.

## 3. Results

### 3.1. Sample Size

A total of 100 freshly culled Eastern Grey Kangaroos (90 adult and 10 juveniles; 65 male and 35 female) were sampled within 4 h of culling.

### 3.2. Bacteria Characterisation

MSSA and MRSA were isolated from 13 nasal and 6 rectal samples, respectively, with 2 kangaroos colonised with MRSA in both nasal and rectal passages. MSSA grew with another pathogenic organism in 62% (8/13) of these positive nasal samples, with *S. sciuri* comprising 75% (6/8) of these co-colonised samples (Table 1).

The most commonly isolated bacterium with pathogenic potential was *Pseudomonas putida* (*P. putida*), which was mainly present in rectal samples, with 2 animals carrying the bacteria in both nasal and rectal flora (28% of kangaroos; 27/100 of rectal samples, 3/100 of nasal samples). CoNS was present in 17% of kangaroo nasal isolates and 41% of these also harboured *Mammaliicoccus sciuri* (*M. sciuri*). The rectal passages did not isolate any CoNS.

*Staphylococcus haemolyticus* was found in 10% of nasal isolates and *M. sciuri* in 7%. Other potentially pathogenic bacteria were found in 5% or less of both nasal and rectal samples. Ten nasal samples demonstrated clustered growth of *Staphylococcus saprophyticus* (*S. saprophyticus*), *Staphylococcus haemolyticus* (*S. haemolyticus*), *Staphylococcus warneri* (*S. warneri*), *Micrococcus*, and *Streptococcus* B/D together, with one sample additionally growing *Stenotrophomonas maltophilia* (*S. maltophilia*).

### 3.3. Comparative Analysis

In response to an increased incidence of SSTIs in military recruits undergoing basic training, Suhr et al. conducted a prospective observational cohort study to investigate SSTI incidence and *S. aureus* carriage rates over a 16-week period [14]. They found a significant increase in *S. aureus* colonisation from the commencement to the conclusion of training (19% to 49%), consistent with previous studies of similar populations [12,18,19,20,21,22,23]. Samples from the nares and rectum from the same Eastern Grey population studied by Suhr et al. were taken for this study. The previous study discovered that the majority (77%) of participants reported skin trauma during their training with nearly 20% requiring antibiotics. Of the wound swabs that were taken, 42% grew MRSA and 33% grew *S. aureus*. The researchers found that a total of 83% of environmental samples demonstrated a *Staphylococcal* spp. with over a third (36%) discovering *S. aureus.* After 1-night of living on base, 30% of mattresses and bed linen were colonised by *S. aureus*. Comparative antibiograms suggest human to human transmission. At this same training site, our samples demonstrated that the same bacteria were harboured by Eastern Grey Kangaroos at a similar pre-training rate of new military recruits. Additionally, multiple pathogenic bacterial species were frequently detected within the same sample, indicating the potential for polymicrobial infection. This suggests that these marsupials can spread pathogenic bacteria to the environment shared by humans. Further spread of the pathogen into human living environments, and subsequently human to human transmission, occurs rapidly indicating that personal hygiene, particularly around times of outdoor activity, are likely to be significant in preventing SSTIs.

## 4. Discussion and Conclusions

Eastern Grey Kangaroos are widespread across the eastern and southeastern regions of Australia and are known to serve as reservoirs for *Coxiella burnetti* (Q Fever), *Leptospira* spp. (Leptospirosis), Giardia (Giardiasis), and *Cryptosporidium* spp. (Cryptosporidiosis). Previous studies have not investigated carriage rates of potentially human pathogenic bacteria from direct sampling of free-ranging kangaroos and much of the literature involving animal *Staphylococcal* carriage is limited to studies of captive or domesticated animals which had regular contact with humans [16,17,24]. The scheduled kangaroo cull provided an opportunity to collect fresh nasal and rectal samples (obtained less than 4 h post cull) to determine the characteristics and prevalence of bacteria with pathogenic potential to humans. This is the first study to investigate the prevalence of pathogenic bacteria in an isolated, free-ranging Eastern Grey Kangaroos inhabiting a shared environment with humans. It was particularly significant in this study to identify and isolate coagulase-negative Staphylococci (CoNS) in clusters. Though clinically less pathogenic than *S. aureus*, CoNS have been emerging as important in immunocompromised patients and patients with foreign material in situ [25]. CoNS serve as a reservoir for antibiotic-resistant genes as they are frequently found on mobile genetic elements [26]. In this study, CoNS was found in 17% of nasal samples indicating that this route can serve as a reservoir for the transfer of antibiotic-resistant genes.

*S. saprophyticus* is a CoNS that colonises the perineum, rectum, urethra, cervix, and gastrointestinal tract in humans, and is a common cause of uncomplicated UTIs in young, sexually active females [25]. *S. saprophyticus* has become increasingly resistant to multiple antibiotics in the community setting [27]. *S. saprophyticus* was found in 10% of nasal samples in the present study and was exclusively grown with other CoNS with human pathogenic potential. The co-colonisation of *S. saprophyticus* with other CoNS, such as *S. haemolyticus* and *S. warneri*, as discovered by this study, suggests the potential of *S. saprophyticus* to inherit resistance or virulence genes possessed by other CoNS through mechanisms such as through horizontal gene transfer, mobile genetic elements, biofilm formation, or selective pressure [25,26]. An interesting finding from this study was that *S. haemolyticus*, *S. saprophyticus*, *S. warneri*, *and Micrococcus*, and *Streptococcus* B/D did not grow independently from each other, suggesting a microenvironment favouring their combined growth or a symbiotic mechanism within the nasal cavity of Eastern Grey Kangaroos.

*M. sciuri*, which inherently possesses the ability to transfer resistance genes to other staphylococcal species [28,29,30], was the only pathogenic CoNS to grow with MSSA or MRSA, though in low levels, 6% and 1%, respectively. MSSA grew with another human pathogenic organism in 62% (8/13) of nasal samples, with *M. scirui* found in 75% (6/8) of these samples. This finding is significant because *M. sciuri* is known to encode enterotoxin A and toxic shock syndrome toxin [31]; however, infections caused by *M. sciuri* rarely result in severe disease in immunocompetent humans. Additionally, *M. sciuri* is known to harbour the *mecA* gene [30,31] which encodes for an altered penicillin binding protein (PBP2a) allowing for cell wall biosynthesis in the presence of most beta-lactam antibiotics, representing the key determinant of MRSA resistance. A growing body of evidence suggests that *M.* sciuri is the ancestral reservoir of the *mecA* gene found in clinically significant MRSA strains. The *mecA* gene in MRSA exhibits a high degree of homology at the protein sequence level with *M. sciuri* suggesting an evolutionary link that has likely contributed to the contemporary emergence and dissemination of MRSA. Furthermore, *M. sciuri* isolates carrying the *mecA* gene have been discovered in a diverse range of sources, including soil, as well as the skin and mucous membranes of wild animals, highlighting its ecological versatility in ongoing environmental persistence and resulting in continued gene transmission [32]. The potential for less pathogenic bacteria to facilitate the acquisition of resistance genes to more pathogenic bacteria may lead to the emergence of difficult to treat, invasive infections.

The findings in our study suggest a potential for the transfer of genes, which can result in an increased virulence or antibiotic-resistant Staphylococcal species. This is particularly significant in human populations who live in close living quarters and share an environment with animals. Most of the other potentially pathogenic organisms detected in the nasal, perianal, and rectal regions in this Eastern Grey Kangaroo population were discovered in low levels and are less likely to be clinically relevant as a direct source of infection, as these bacteria are typically associated with rare, opportunistic nosocomial infections in severely immunocompromised patients. Bacterial carriage evaluation of this kangaroo population, which was present during the investigation of recent incidence of nMRSA SSTI in military recruits demonstrates the interconnection between humans, kangaroos, and their shared environment. The results of this study indicate that Eastern Grey Kangaroos can harbour pathogenic bacteria with the ability to transmit resistance genes. Future studies are planned to further compare genomic sequencing to better understand transmission and investigate virulent genes.

## Figures and Tables

**Table 1:** Growth of Known Pathogens from Rectal and Nasal Swabs of *Macropus giganteus*.

Bacteria	Number of Kangaroos with Isolates from Rectal Swabs (n = 100)	Number of Kangaroos with Isolates from Nasal Swabs (n = 100)	Main Clinical Significance
MSSA	6	13	Common cause of a wide array of both community-acquired and hospital-acquired infections. Ubiquitous colonisation of human flora.
MRSA	2	4	Causes a wide array of infections including SSTIs, bone infections, joint infections, endocarditis pneumonia, and bacteraemia. Associated with significant morbidity and mortality. One of the most common causes of hospital-acquired infections. Resistant to beta-lactam antibiotics.
*Staphylococcus saprophyticus*	0	10	Most common cause of uncomplicated urinary tract infections (UTI) in young, sexually active females.
*Staphylococcus haemolyticus*	0	10	Common cause of infections of the central nervous system, diabetic foot ulcer infections, and is a common pathogen in patients with neurosurgical procedures. Often an opportunistic infection. Increasingly becoming multidrug-resistant. Carries resistance genes and is a known reservoir for distribution of antimicrobial resistance staphylococci
*Staphylococcus warneri*	0	10	Rare cause of UTI, rare cause of sepsis in immunocompromised patients. High degree of antimicrobial resistance.
*Pseudomonas aeruginosa*	0	1	Can result in a wide array of infections. Commonly found in immunocompromised patients and typically multidrug-resistant.
*Pseudomonas putida*	27	3	Rarely results in infection. Found to cause infection in patients undergoing invasive procedures or in immunocompromised patients.
*Pseudomonas fluorescens*	2	0	Wide range of infections in immunocompromised patients. Many resistant phenotypes have been characterised. May play a role as a reservoir for beta-lactamase resistance genes or other antimicrobial resistance genes.
*Streptococcus* *agalactiae* (Group B *Streptococcus*; GBS) or Group D *Streptococcus*	0	10	GBS is the leading cause of postpartum infection and neonatal sepsis. Can be vertically transmitted to neonates. GBS is known to cause SSTIs in patients with diabetes and is linked to heart failure in the elderly population. Group D *Streptococcus* is known to cause bacteraemia and subsequent endocarditis. *Streptococcus bovis* is associated with gastrointestinal malignancies
*Mammaliicoccus sciuri* (formerly *Staphylococcus sciuri)*	0	7	Rare cause of toxic shock syndrome. Potential reservoir for virulence and resistance genes.
*Acinetobacter haemolyticus*	1	0	Rare cause of nosocomial infections, mainly aspiration pneumonia and catheter-associated bacteraemia but known to cause UTIs and SSTIs.
*Bacillus cereus group*	0	1	Foodborne pathogen resulting in emetic and diarrheal syndromes. Can cause SSTI with penetrating trauma or mucosal injury.
*Stenotrophomonas maltophilia*	3	1	Newly emerging pathogen of concern. Intrinsically multidrug-resistant. Opportunistic, often respiratory infection, associated with high morbidity and mortality in immunocompromised patients. Also causes bacteraemia and line- associated infections. Less commonly associated with SSTIs.
*Stenotrophomonas* *rhizophila*	1	0	Intrinsically multidrug-resistant, mainly opportunistic infections.
*Enterobacter cloacae complex*	1	0	Commonly associated with nosocomial infections resulting in a wide array of clinical consequences and is becoming increasingly antimicrobial-resistant.
*Enterococcus casseliflavus*	0	1	Nosocomial infections, most commonly associated with bacteraemia and trauma-induced endophthalmitis, but also known to cause surgical site infections, UTIs, and infective endocarditis. Carries the *VanC* gene, conferring resistance to vancomycin.
*Pantoea agglomerans*	5	0	Rare cause of opportunistic wound infection, mostly found in immunocompromised patients.
*Pseudomonas oryzihabitans*	1	0	Rare cause of nosocomial infections. Hospital outbreaks described in the literature.
*Microbacterium* *arborescens*	0	2	Rare cause of infection in immunocompromised patients.
*Curtobacterium flaccumfaciens*	0	1	Case reports of opportunistic infections. Considered to be lowly pathogenic.
*Dermacoccus nishinomiyaensis*	1	0	Generally not considered a human pathogen. Case reports of opportunistic infections.
*Micrococcus*	0	10	Generally not considered a human pathogen. Case reports of opportunistic infections.

## Data Availability

The data presented in this study are available on request from the corresponding author due to Australian National Defence related privacy and controlled data accessibility.
